# Condition-Dependent Neural Dimensions Progressively Shift during Reach to Grasp

**DOI:** 10.1016/j.celrep.2018.11.057

**Published:** 2018-12-11

**Authors:** Adam G. Rouse, Marc H. Schieber

**Affiliations:** 1 Department of Neuroscience, University of Rochester, Rochester, NY 14642, USA; 2 Department of Neurology, University of Rochester, Rochester, NY 14642, USA; 3 Department of Biomedical Engineering, University of Rochester, Rochester, NY 14642, USA; 4 Del Monte Institute for Neuroscience, University of Rochester, Rochester, NY 14642, USA; 5 Lead Contact

## Abstract

Neural population space analysis was performed to assess the dimensionality and dynamics of the neural population in the primary motor cortex (M1) during a reach-grasp-manipulation task in which both the reach location and the object being grasped were varied. We partitioned neural activity into three components: (1) general task-related activity independent of location and object, (2) location- and/or object-related activity, and (3) noise. Neural modulation related to location and/or object was only one-third the size of either general task modulation or noise. The neural dimensions of location and/or object-related activity overlapped with both the general task and noise dimensions. Rather than large amplitude modulation in a fixed set of dimensions, the active dimensions of location and/or object modulation shifted progressively over the time course of a trial.

## In Brief

Rouse and Schieber show that during reach-grasp-manipulate movements, M1 activity related to location and object occurs not in a fixed set but rather in a shifting set of neural dimensions that overlap with those of general task and noise activity.

## Graphical Abstract

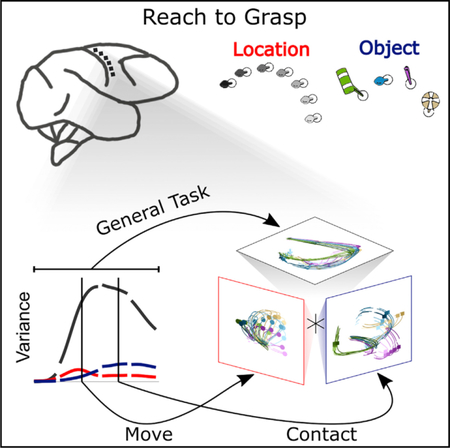

## INTRODUCTION

Descending signals from the brain generate coordinated movements such as reaching, grasping, and manipulating. These signals would be potentially straightforward if some neurons were responsible for signaling when to move, others where to reach, and yet others what to grasp and manipulate. In the primary motor cortex (M1) during a reach-grasp-manipulate (RGM) task, however, we observed that individual neurons do not appear to segregate into such specific groups (Rouse and Schieber, 2016a). In particular, the firing rate of most M1 neurons varied in relation both to reach location and to the object being grasped and manipulated. We now examine how much of the neural variance in the M1 population is related to the general action of per-forming an RGM movement versus the specific aspects required for a particular object in a particular location. We challenge the hypothesis that the neural variance related to the task in general, to location and/or object feature tuning, and to noise each occur in a separate subspace. We also test the hypothesis that location and/or object encoding occurs in a fixed set of neural dimensions that remains stable as a movement trial proceeds in time.

Variation in the firing rate of neurons in the M1 during an experimental task can be viewed as having three major parts: task-specific encoding, condition-independent modulation, and noise. First, the firing rate of M1 neurons varies in relation to specific parameters or other features of bodily movement, such as kinematics, kinetics, muscle activity, etc. Such relation-ships, often referred to as tuning or encoding, have been demonstrated in relation to movement direction and velocity (Georgopoulos et al., 1986; Moran and Schwartz, 1999), trajectory fragments (Hatsopoulos et al., 2007), force (Kalaska et al., 1989; Fagg et al., 2009), electromyographic (EMG) activity in particular muscles (Thach, 1978; Pohlmeyer et al., 2007), combi-nations of movement kinematics and muscles (Kakei et al., 1999; Griffin et al., 2015), and movement of particular fingers (Schieber and Hibbard, 1993). As movements progress in time, these features can be decoded from populations of simultaneously recorded M1 neurons (Georgopoulos et al., 1988; Salinas and Abbott, 1994; Wessberg et al., 2000).

A second part of firing rate variance in M1 neurons reflects the structure of the behavioral task used in such studies, which typically involves a movement preceded and followed by maintained postures. During the movement, the firing rate of many M1 neurons modulates relative to their individual baselines regardless of the particular experimental conditions in the cur-rent trial. The firing rates of most M1 neurons are comparatively low until ~ 100 ms before the onset of movement, change during the movement, and finally return to near baseline levels as a final posture is established and maintained (Fortier et al., 1993; Crammond and Kalaska, 2000; Velliste et al., 2014). Although the discharge of some individual neurons may decrease for some or all of the experimental conditions, the majority of M1 neurons and population averages both typically show a general increase in activity during movement (Rouse and Schieber, 2016a). Such a general increase in the discharge of M1 neurons during movement contrasts both with that of spinal interneurons, which tend to show increased discharge during movement that is maintained while the final posture is held (Shalit et al., 2012), and with that of cerebellar Purkinje cells and nuclear neurons, which have a substantial tonic discharge rate that can be modulated both higher and lower during movement (Thach, 1970a, 1970b).

A third part of firing rate variance in M1 neurons, like that of many other CNS neurons, is noise. Some of this noise may reflect variation in neural signals related to features not controlled in the current experiment, which becomes evident in the shared noise correlations among a population of M1 neurons (Lee et al., 1998; Goris et al., 2014; Vinci et al., 2016). Noise also may include a stochastic component that reflects individual neuron variability in the cellular processes underlying spike generation (see, however, Mainen and Sejnowski, 1995).

The firing rates of individual M1 neurons typically include all three parts (Rouse and Schieber, 2016a). Nevertheless, task-specific signals, condition-independent signals, and noise each might be ascribed to different linear or even non-linear combinations of the firing rates of the simultaneously recorded neural population, which we will refer to as neural dimensions (Yu et al., 2009; Cunningham and Yu, 2014; Sadtler et al., 2014; Kobak et al., 2016). A recent analysis of activity in the dorsal premotor and M1 during reaching, for example, found that reaching-tuned activity occurred in neural dimensions that were largely orthogonal to those of the simultaneous condition-invariant activity (Kaufman et al., 2016). To what extent might such be the case for more complex movements that involve not only reaching but grasping and manipulation as well? Here, using the activity of M1 neuronal populations recorded simultaneously during movements that involved reaching, grasping, and manipulation, we tested the hypothesis that these three partitions of neural variance—related to location and/or object feature tuning, to the task in general, and to noise—are segregated into three separate, orthogonal subspaces of the neural space. In addition, we tested the hypothesis that as a trial progresses in time, location and/or object encoding remains fixed within a small number of neural dimensions.

## RESULTS

We analyzed neural activity recorded as two monkeys performed an RGM task involving 4 different objects each positioned at up to 8 different locations, described in detail previously (Rouse and Schieber, 2015, 2016a). For the present study, we selected only those recording sessions in which data had been collected simultaneously from all 6 microelectrode arrays implanted in M1, spanning the entire upper extremity representation of “new” M1 (Rathelot and Strick, 2009) in the anterior bank and lip of the central sulcus. We analyzed the firing rates of all recorded units from all electrodes, totaling N = 346 M1 units from monkey L and N = 466 M1 units recorded from monkey X

For each unit, data were available from approximately 30 successfully completed trials involving each of 24 different location and/or object conditions (see STAR Methods). In populations of M1 neurons recorded simultaneously during RGM movements, we first partitioned the variance of M1 firing rates that was related to (1) the execution of a movement in general, (2) the two independent variables of the behavioral task—the location to which the subject reached and the object the subject grasped and manipulated—and (3) the remaining noise (see STAR Methods; Figure 7). We used this partitioned data to determine whether neural dimensions of location and/or object encoding occurred in a separate subspace orthogonal to that of general task activity and/or noise. We also examined whether the location and/or object encoding subspace shifted with time during a trial.

### Variance in Partitioned Neural Activity

**Figure 1.**
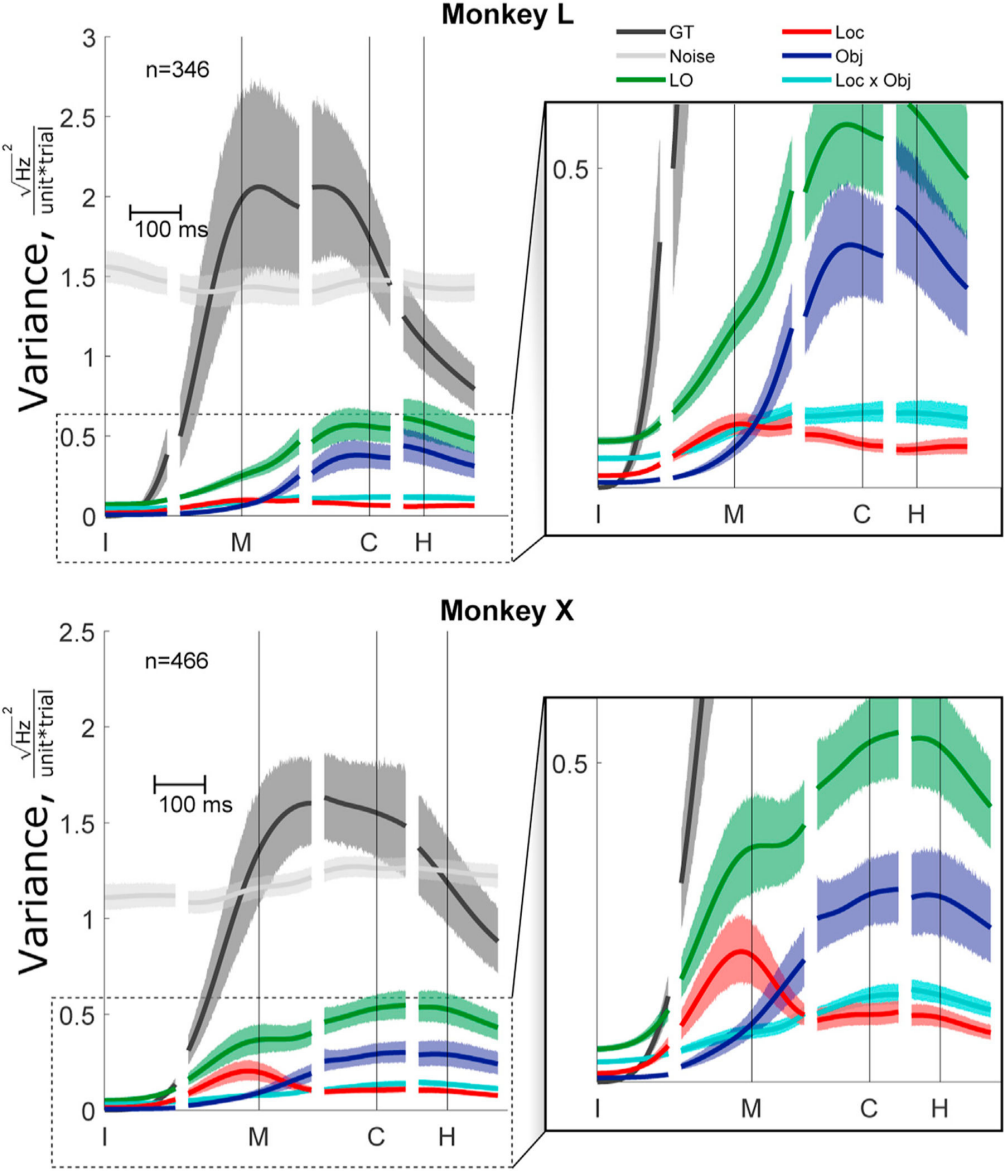
Time-Resolved Variance of Neural Firing Rate across the Recorded Population Partitioned by General Task (Black), Location and/or Object (Green), and Noise (Gray) The location and/or object variance is further sub-divided by location (red), object (blue), and location 3 object interaction (cyan). The right panel is an enlargement to enhance visualization of the time course of the location and object partitions. The variance is calculated as the sum of squares of the square-root transformed firing rate and normalized by dividing by the number of units and number of trials resulting in the squared firing rate per unit per trial. Data have been aligned separately on the times of instruction (I), movement onset (M), contact (C), and hold (H). Shaded regions for each curve show the 95% confidence interval for the mean variance of that partition (see STAR Methods).

We initially compared the variance in each partition averaged across all units as a function of time in RGM trials. Figure 1 illustrates the time course of the general task (GT), location and/or object (LO), and noise sample variance, averaged across all trials in each monkey. GT variance is the result of each neuron’s firing rate averaged across all task-specific conditions changing relative to the individual neuron’s baseline. In both monkeys, the GT variance (black) started at zero (see STAR Methods) and increased as neurons modulated away from their individual baselines, becoming largest between movement onset (M) and peripheral object contact (C) and decreasing thereafter. Most units increased their firing rates relative to baseline, as follows: 82% (282/346) for monkey L and 78% (364/466) for monkey X (Figure S1). The LO variance (green) began to increase at about the same time, but peaked shortly after peripheral object contact, continually remaining substantially smaller than the GT variance. In contrast to the GT and the LO variance, the noise variance (gray) remained relatively constant over the time course of RGM trials. Noise variance was continually larger than LO variance.

We further subdivided the LO partition into variance related to location, to object, and to location × object interactions. Across all times, 185/346 = 53% (L) and 298/466 = 64% (X) of all the re-corded units were tuned to location, 251/346 = 73% (L) and 391/ 466 = 82% (X) were tuned to object, and 177/346 = 51% (L) and 284/466 = 61% (X) were tuned to both location and object (Figure S2). As shown in Figure 1 (enlargements), however, the time course of location-related variance (red) and object-related variance (blue) did not simply follow that of the collective LO variance. Though more overt in monkey X than in monkey L, location-related variance peaked earlier near the time of movement onset (M), while object-related variance peaked later near the time of peripheral object contact (C), with the interaction-related variance remaining relatively small throughout (Rouse and Schieber, 2016a). Significant location-related modulation was present at movement onset in 86/346 (25%) units in monkey L and 137/466 (29%) units in monkey X and at peripheral object contact in 66/346 (19%) in L and 146/466 (31%) in X. Conversely, object-related modulation was present at movement onset in 72/346 (21%) in L and 126/466 (27%) in X, increasing by the time of peripheral object contact to 186/346 (54%) in L and 293/466 (63%) in X.

To quantify the magnitude of variance in the three partitions, we averaged the square-root firing rate variance per unit per trial across all time points. The average variance in the GT partition was 1.27 (95% confidence interval [CI]: 1.01–1.53) in monkey L and 1.01 (95% confidence interval: 0.87–1.16) in monkey X, in the LO partition was 0.36 (0.31–0.41) in L and 0.35 (0.31–0.40) in X, and in the noise partition was 1.45 (1.37–1.54) in L and 1.19 (1.14–1.24) in X. In each monkey, the average neural variance in the GT and noise partitions was similar in magnitude, whereas the average neural variance in the LO partition was only one-third as much. Within the LO partition, the average location-related variance was 0.07 (0.06–0.08) in monkey L and 0.10 (0.09–0.12) in monkey X, the average object-related variance was 0.20 (0.16–0.24) in L and 0.16 (0.13–0.19) in X, and the location × object interaction variance was 0.09 (0.08–0.10) in L and 0.09 (0.08–0.10) in X. In both monkeys, the neural variance in the LO partition thus was significantly less than the variance in both the GT partition and the noise partition. Within the LO partition, object-related variance was significantly larger than location-related variance.

### Dimensionality of Neural Partitions

**Figure 2.**
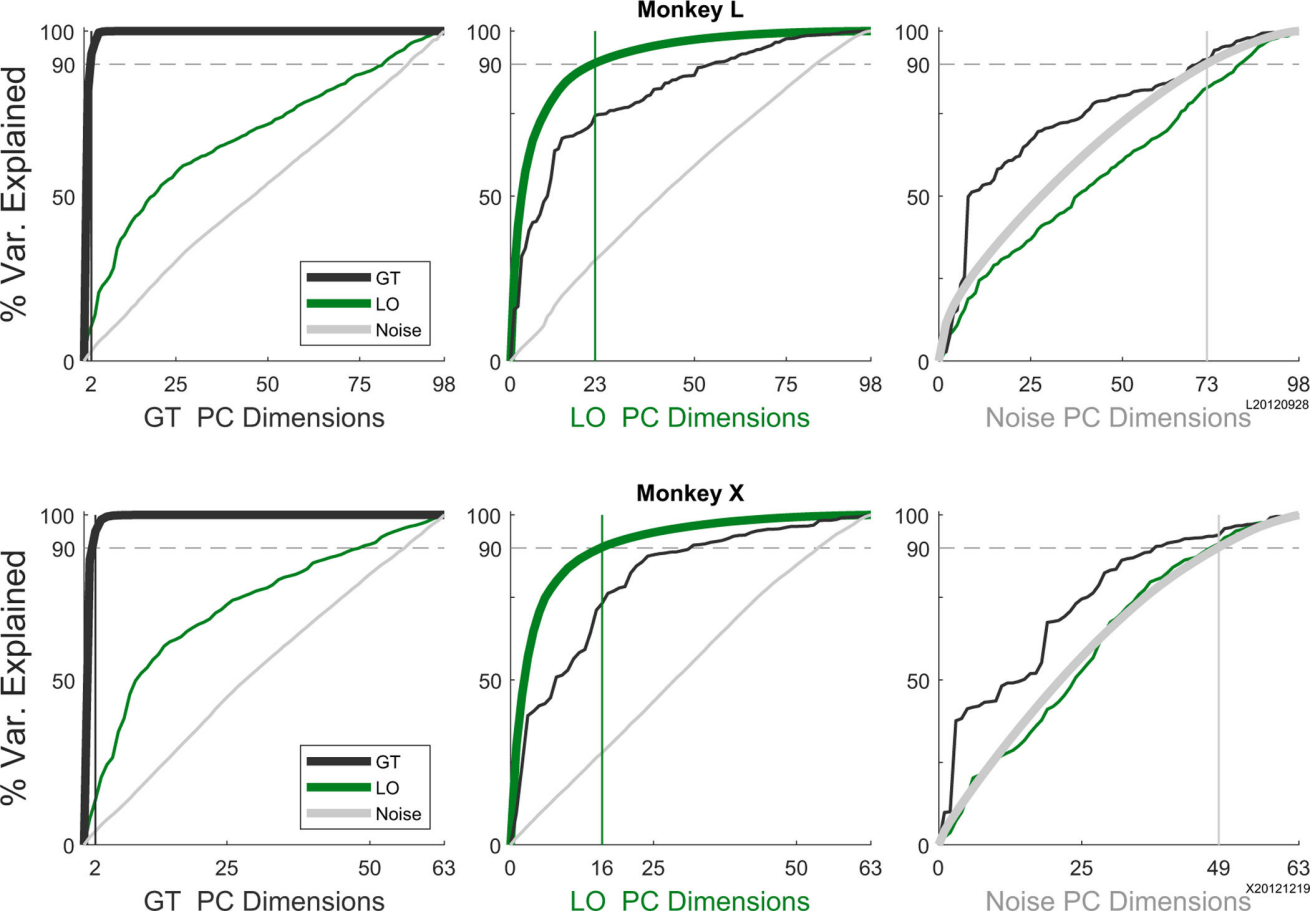
Dimensionality and Overlap of Neural Activity Partitions PCA was performed separately on each of the 3 partitions. In each panel, the thick line shows the cumulative variance explained for data in the partition on which the PCA had been performed; vertical lines indicate the number of dimensions needed to account for 90% of variance in the general task (GT) (left), LO (center), and noise (right) partitions. The cumulative variance explained by those PCs for the data in each of the other two partitions has been plotted as thin lines. Note that because this analysis of noise variability relies on individual trials, the simultaneously recorded data shown here come from a single session.

Having partitioned the firing rate of each unit during each trial, we compared the dimensionality of the neural variance in the three partitions. To identify the neural dimensions that captured most of the variance in each partition, we performed principal component analysis (PCA) separately on that portion of the firing rate modulation of the *N* units related to the GT, the LO conditions, or the noise. Because analysis of the noise required individual trials of simultaneously recorded data, this analysis was performed on a single session with N = 98 units for monkey L and N = 63 units for monkey X. For each monkey, the thick lines in Figure 2 show the cumulative variance explained by the rank-ordered principal components (PCs) of the GT activity (left, black), the LO activity (center, green), and the remaining noise (right, gray). In each monkey, ≥ 90% of the GT variance was explained by the first 2 PCs (left, vertical black line), and virtually all (≥99%) was explained by the first 4 PCs, indicating that this condition-independent modulation of all *N* units occurred in a low-dimensional subspace of the *N*-dimensional neural space. The dimensionality of the LO modulation was higher. To account for ≥ 90% of this condition-dependent variance required 23 PCs in monkey L and 16 in monkey X (center, green vertical line). The dimensionality of the noise partition was higher still, requiring 73 PCs in L and 49 in X to account for ≥ 90% of the variance. Noise modulation thus was distributed relatively evenly across the population of units in each mon-key. Relatively uniform noise standard deviations across units and low magnitude pairwise noise correlations suggested that the activity in the noise partition was consistent with a uniform, independent noise model. (Figure S3). The differences observed in the dimensionality of the three partitions did not result from GT or LO modulation occurring in limited numbers of units but rather represent common, correlated modulation occurring across the M1 population (Figure S4). Within the LO partition, rather than location- and object-related variance occurring in different units, the units with large location-related variance were more likely to have considerable object-related variance, and conversely those with large object-related variance were more likely to have considerable location-related variance (Figure S5).

To what extent did the neural dimensions that captured most of the GT variance also capture LO variance and vice versa? To address this question, we also examined the cumulative variance of each of the other two partitions projected onto the rank-ordered PCs determined for a given partition, shown as thin lines in Figure 2. The 2 PCs that captured ≥ 90% of the GT variance captured only 11% (L) or 14% (X) of the LO variance, indicating that most of the LO modulation occurred outside of the GT subspace. In contrast, the 16 dimensions that explained ≥ 90% of the LO variance also captured 74% (L) or 73% (X) of the GT variance. The noise variance projected onto either the GT PCs or the LO PCs increased as a relatively linear function of the number of PCs, indicating that the noise variance was distributed evenly across the population of units in each monkey, unrelated to the dimensions containing GT and/or LO activity.

For each monkey, we also quantified the overlap between the variance distributions of all three possible pairs of partitions. Overlap was measured using a normalized sum of dot products across all neural dimensions weighted by the variance in each dimension (see STAR Methods). A 95% confidence interval was also estimated as if the two variance distributions had been oriented at random. The following overlap between GT variance and the LO variance was somewhat more than could be expected by chance alone: 0.26 (chance 95% confidence interval: 0.02–0.18) in monkey L and 0.26 (0.02–0.20) in monkey X. In neither monkey were these variance distributions orthogonal to one another (overlap = 0). The overlap between GT and noise was 0.18 (0.11–0.17) and 0.24 (0.15–0.21) for monkeys L and X, respectively. The overlap between LO and noise was 0.29 (0.20–0.29) and 0.36 (0.26–0.33) for monkeys L and X. Over-lap of the noise partition with either the GT or LO partition was similar to or slightly greater than what would be expected by chance alone. Noise dimensions were not orthogonal to either GT or LO dimensions.

### Neural Trajectories during RGM Movements

**Figure 3.**
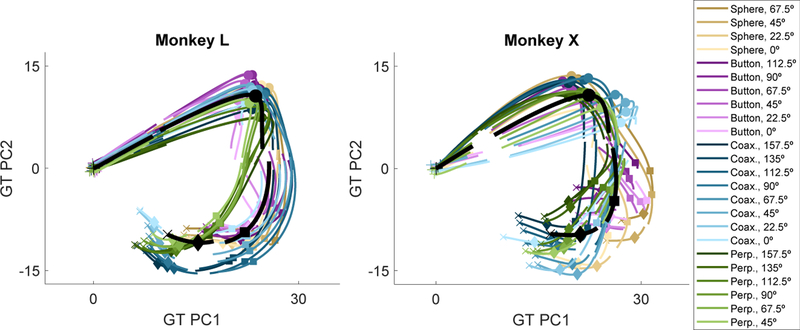
Neural Trajectories through a GT-Derived PC Space for Monkeys L and X The neural trajectory for each of the 24 location and/or object conditions (colored lines) as well as the GT (black line, averaged across all trials) has been projected onto the first 2 PCs derived from the GT partition, using the data concatenated across all trials of all locations/object conditions at all times for each monkey. In the color scale, saturation represents location (0°, lightest; 157.5°, darkest), whereas hue represents object (sphere, tan; button, purple; coaxial cylinder, blue; perpendicular cylin-der, green). Data have been aligned separately at the times of instruction (+), movement onset (circle), contact (square), and hold (diamond).

The firing rates of all *N* simultaneously recorded units can be considered instantaneously as a single point in an *N*-dimensional neural state space, and the time series of successive points then forms a neural trajectory through this space. We first examined such neural trajectories during RGM trials by performing PCA on the GT partition of neural activity. The black traces in Figure 3 show the GT trajectory for each monkey projected in the plane of the first two GT PCs. In addition, separate curves representing the neural trajectories of the summed GT + LO activity for each of the 24 LO conditions (averaged across trials) have been projected onto these first two GT PCs. Rotational dynamics were evident in the overall GT trajectory. Proceeding clockwise in time, the black GT trajectory begins at instruction onset (+), pro-gresses to the onset of movement (filled circles), rotates sharply and then proceeds downward to the time of peripheral object contact (filled squares), turns again through completion of the manipulation (filled diamonds), and ends 100 ms later during the final hold (x). The 24 different colored GT + LO trajectories generally follow the overall GT rotation, deviating from it to different degrees at different times and changing in their posi-tions relative to one another.

### Time-Resolved PCA of the LO Partition

We next performed PCA on the LO partition alone and then pro-jected the 24 LO trajectories (without GT activity) in the first six LO PCs (Figure S6). These trajectories showed no simple organi-zation. The maximal separation of different locations/object tra-jectories in this LO subspace occurred at different points in time, suggesting that the neural dimensions of the LO partition may shift progressively over the time course of RGM trials.

**Figure 4.**
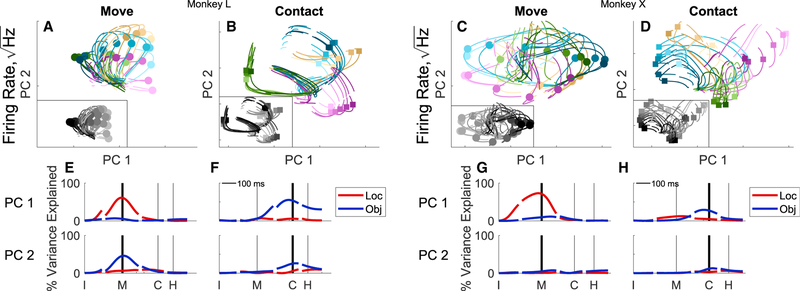
Time-Specific PCs of the Location and/or Object Partition For each monkey (L, left; X, right), PCs were derived from the data at two particular time points, as follows: movement onset and peripheral object contact. The trajectories for each of the 24 location and/or object combinations then have been projected into the plane of the first two move PCs (A and C) and the first two contact PCs (B and D), respectively. Colors are the same as Figure 3 with insets showing gray scale to emphasize location (lightest, 0; darkest, 157.5). Location, object, and location 3 object variance are shown as functions of time for PC1 (upper row) and in PC2 (lower row) of the move subspace (E and G) and of the contact subspace (F and H).

To examine this possibility further, we performed PCA on the partitioned LO activity taken at two separate time points—move-ment onset (M) and peripheral object contact (C)—and then pro-jected the neural trajectories of the 24 different LO conditions onto the first two PCs derived at movement onset and separately onto the first two PCs derived at peripheral object contact (Figure 4, top). More specifically, for each neuron we averaged the firing rate separately at the movement onset and at the periph-eral object contact time points across all trials of each of the 24 conditions and then performed PCA on these averages for all the units from a given monkey. We refer to these two sub-spaces as “move” and “contact,” respectively. We then pro-jected the averaged firing rates for each of the 24 conditions at all time points onto the first two PCs of the move subspace and the contact subspace separately.

The same high-dimensional neural trajectories projected into these two different PC spaces were quite different in each monkey. Projected onto the first two PCs of the move subspace, the trajectories diverged substantially near the time of movement onset (circles) based largely on location (Figures 4A and 4C, color saturation scale, emphasized by the gray scale inset). In contrast, when projected onto first two PCs of the Contact subspace the trajectories diverged most near that time point (squares) largely depending on the object (Figures 4B and 4D, color hue).

We quantified this apparent difference in location versus ob-ject separation of the 24 trajectories in the move versus contact subspaces by calculating at each time point the location vari-ance and the object variance explained by PC1 and by PC2 as a fraction of the total LO variance across all dimensions. Although the fraction of location versus object variance differed between monkeys, in both monkeys the fractional location- related variance of PC1 was greatest near the time of movement onset (M; Figures 4E and 4G), whereas the fractional object-related variance was greatest near the time of peripheral object contact (C; Figures 4F and 4H). PC2 captured a smaller fraction of object-related variance in both subspaces. At time points further from the movement onset or peripheral object contact, these time-specific PC subspaces captured less of the location and object variance. These differences in the move versus contact subspaces provide additional evidence that the neural dimensions of the LO partition shift over the time course of RGM trials.

In previous studies of reaching movements, jPCA (Churchland et al., 2012) has shown strong rotational dynamics of condition-dependent activity (similar to our LO partition), with different neural trajectories all rotating at different radii around a common center. Common rotation was less evident in the present LO neural trajectories in the move and contact PCA spaces. We therefore performed jPCA on the present LO partition from each monkey. As shown in Figure S7, the LO trajectories in the jPCA space, while showing some degree of rotational dynamics, were complex and less simply organized for RGM movements than for the reaching movements of previous studies.

Video S1 illustrates more completely the motion of the 24 LO trajectories projected in the PC1 versus PC2 plane of (1) the GT subspace (left); (2) the move subspace (middle); and (3) the contact subspace (right). All 24 neural trajectories progress through the GT subspace on the left with a similar curved path (Figure 3). Averaged, these 24 GT + LO trajectories form the GT trajectory. Meanwhile, the LO trajectories first separate in the move subspace, with their largest separation reflecting reach location (Figure 4A, saturation). Later, the trajectories separate in the contact subspace, with the largest separation related to the object being grasped and manipulated (Figure 4B, color).

**Figure 5.**
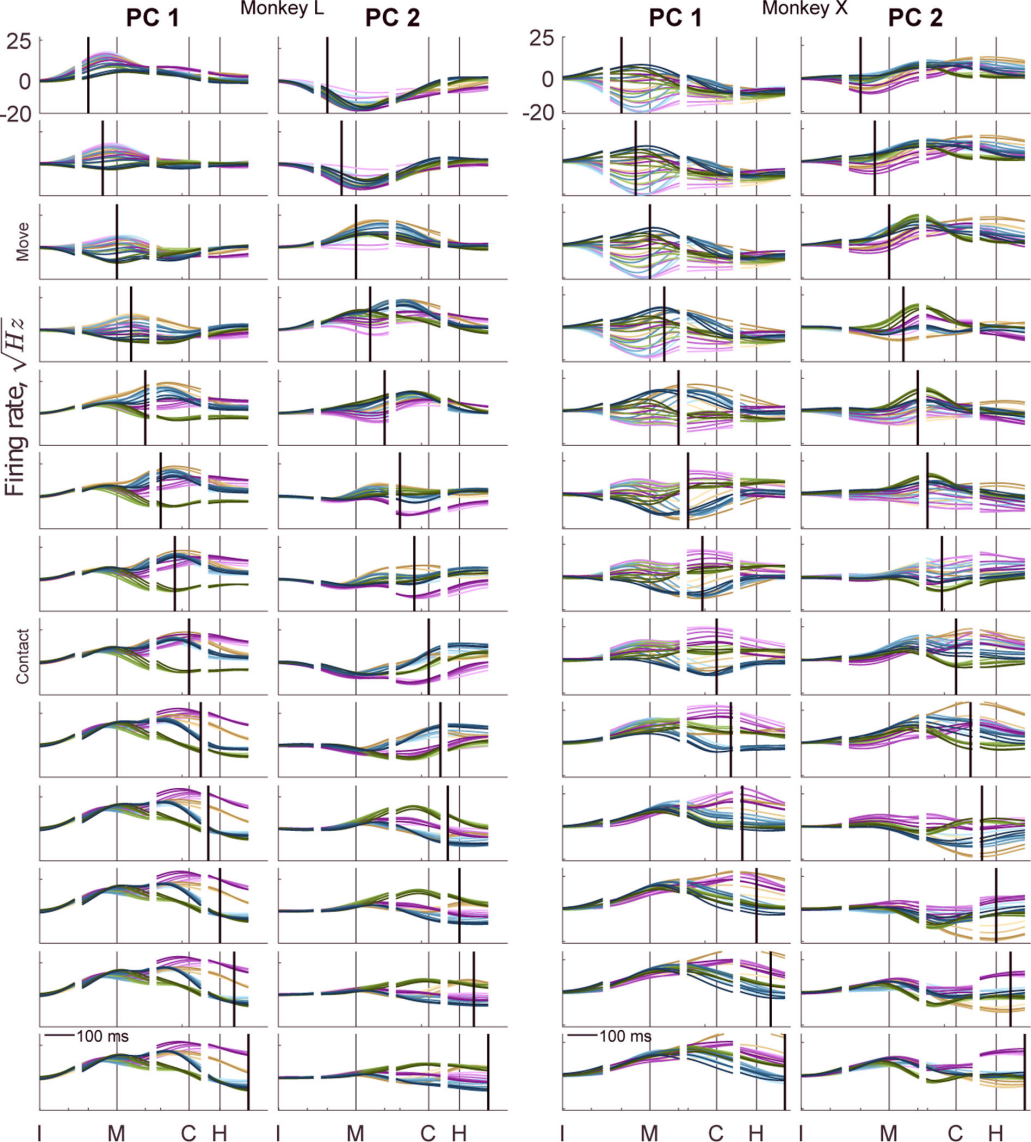
Time-Resolved PCA of LO Activity The24location and/orobject trajectories have been projected as a function of time on to the first two PCs (PC1 and PC2) derived at 13 sequential time points (thick, black vertical lines) progressing sequentially from the top of the figure to the bottom at intervals of50 ms. Move and contact labels indicate the PC subspaces shown in the two-dimensional plots of Figures 4A–4D. Colors as in Figure 3.

These differences in the same neural trajectories projected onto the move versus contact PCs indicate that the neural dimensions capturing much of the LO variance shifted as RGM trials progressed from one time point to another. To examine this progressive shift in greater detail, we performed a PCA using the data at each trial time point separately. Figure 5 shows the 24 high-dimensional LO trajectories projected as a function of time onto the first two PCs of subspaces derived at 13 selected time points. The time point used to derive each set of PCs has been indicated by a thick, black vertical line in each plot. The move and contact subspaces of Figure 4 and Video S1 are indicated at left. In each of these 13 time-specific subspaces, the 24 trajectories dispersed maximally at a time point close to that used to derive the PCs. The maximal dispersion therefore progressed in time, producing a “traveling-wave” appearance. These observations indicate again that within the *N*-dimensional neural state space, the largest variance LO dimensions shifted progressively over the time course of the RGM trials.

**Figure 6.**
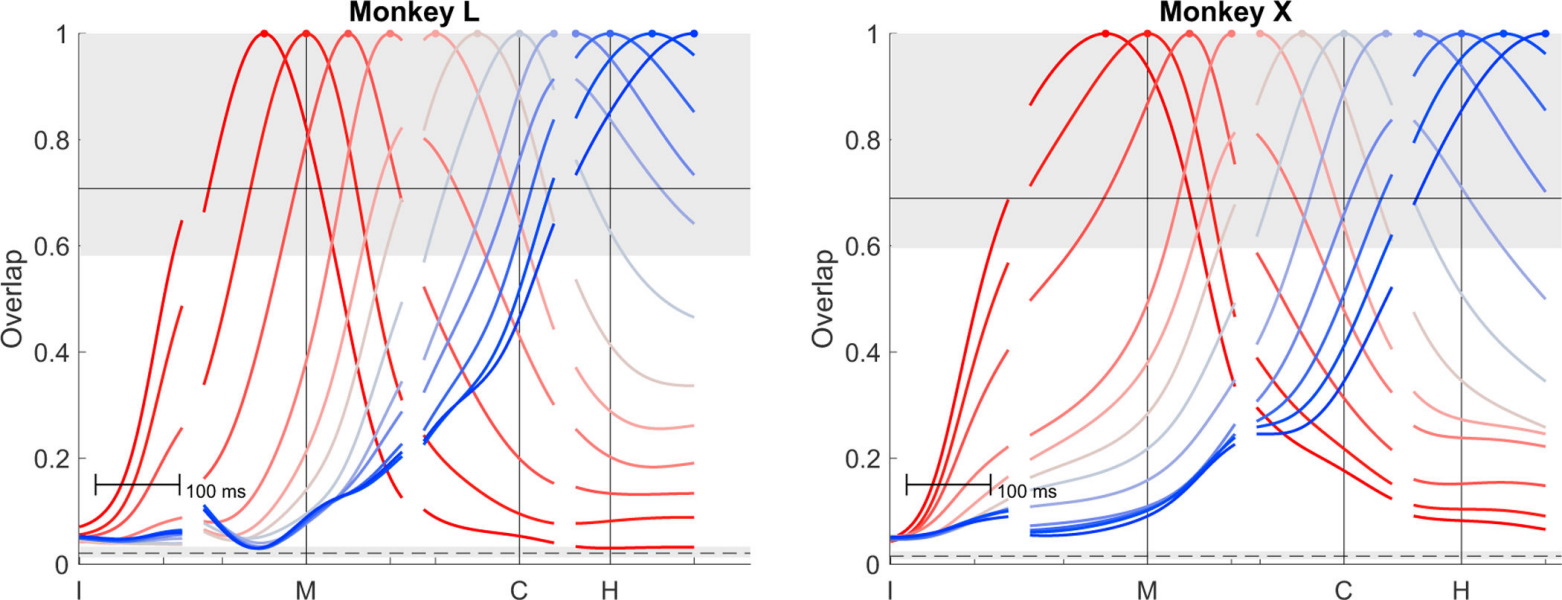
Fractional Overlap of LO PC Subspaces For each of the 13 selected time points illustrated in Figure 5, fractional overlap of the of the LO variance derived at that time point with the LO variance at every other time point is shown as a function of time. The subspace at each selected time point overlapped most with the subspaces at nearby time points and decreased progressively with increasing temporal separation both before and after. Colors are arbitrary and unrelated to colors used in Figures 1, 2, 3, 4, and 5. The range of expected overlaps if two subspaces were sampled randomly from the same fixed, global LO space is shown with the upper shaded region (95% limit) and solid line (median). The range of expected overlaps for two completely unrelated subspaces randomly sampled from an *N*-dimensional space is shown with the lower shaded region (95% limit) and dashed line (median). See STAR Methods for further details.

We quantified the overlap of the LO partition variance derived at different time points by calculating the fractional overlap be-tween the LO variance at each time point compared to the LO variance at every other time point. The overlap was estimated by taking the normalized sum of dot products of all dimensions weighted by each dimension’s variance (see STAR Methods). Figure 6 shows the fractional overlap of LO variance at the 13 selected time points shown in Figure 5 with the LO variance at every other time point, all as a function of time. For each selected time, LO overlap decreased as the temporal separation increased both preceding and following the time point used, consistently producing approximately bell-shaped temporal profiles that plateaued at minimal values for temporal separations beyond ~300 ms in either direction. For statistical comparison (see STAR Methods), we estimated the overlap expected if two randomly sampled subspaces came from either (1) an identical fixed, common LO space sampled twice (upper shaded region) or (2) completely unrelated subspaces sampled independently (lower shaded region). As might have been expected, the degree of overlap for small temporal separations was within the range expected had an identical subspace been sampled twice, whereas the overlap for large temporal separations was not much more than if two subspaces had been selected randomly from unrelated subspaces. These progressive changes in over-lap indicate that the active dimensions of the neural space that encoded location and object information shifted gradually through the global neural space as the trials proceeded in time.

## DISCUSSION

**Figure 7.**
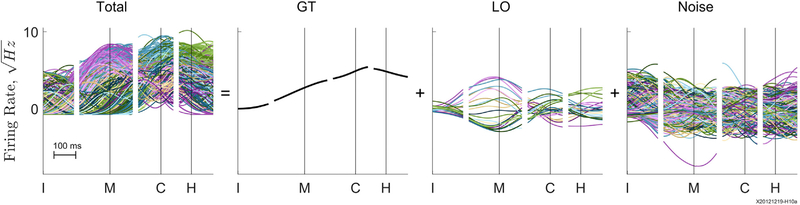
Three Partitions—GT, Location and/or Object, and Noise A single unit’s square-root transformed firing rate during each trial of an experimental session (total) first has been averaged across all trials to obtain the unit’s GT activity. This GT modulation then was subtracted from the original firing rate in each trial, and the remainder was averaged separately for each location and/or object condition (LO). Finally, subtracting both the GT and appropriate LO modulation from the original firing rate left the remaining noise in each trial. Data have been aligned separately at the times of the instruction onset (I), movement onset (M), contact (C), and hold (H). Colors as in Figure 3.

We quantified the variance in populations of M1 neuron firing rates related to the following three factors: (1) the GT, i.e., the performance of a RGM movement trial in general; (2) the particular movement performed, i.e., the combination of the location reached to and the object grasped and manipulated; and (3) noise unexplained by item 1 or 2 (Figure 7). We then compared the neural subspace occupied by these three partitions as well as the neural trajectories in the GT and LO partitions across the time course of RGM trials.

### Variance in the GT, LO, and Noise Partitions

The neural variance in the GT partition was large, indicating substantial changes in neural firing rates during an RGM movement that were similar regardless of the location or object. In part, this GT variance reflects an average increase in discharge during movement relative to posture and may also reflect stronger parametric relationships to movement velocity than to position (Moran and Schwartz, 1999; Reina et al., 2001; Paninski et al., 2004; Saleh et al., 2012). GT variance also may enable transfer of more information from M1 to brainstem and spinal centers (Shalit et al., 2012; Tsianos et al., 2014). Given their tendency for low firing rates during rest, a general increase during movements would enable otherwise quiescent M1 neurons to modulate their firing rates both up and down to encode condition-specific features, such as reach location and grasp shape.

Consistent with a recent report on reaching movements (Kaufman et al., 2016), we observed that the neural variance related to specific LO conditions in our RGM task was substantially smaller than either the GT variance or the noise variance. The smaller magnitude of LO activity contradicts the common impression that firing rate modulation in M1 neurons by and large encodes movement features. Though smaller in magnitude, the LO subspace occupied substantially more neural dimensions than the GT subspace. This higher dimensionality of the LO subspace overlapped partially with the dimensions of the GT subspace that, together with the shift of neural dimensions over the time course of trials (see below), may be key for reliable population encoding of movement features by M1.

The variance of the noise partition was large but was distributed relatively evenly across the entire recorded neural space throughout the duration of movement trials. We found little if any evidence that the neural dimensions with the greatest noise variance were either more or less likely to contain the neural variance related to the GT or LO. These observations support the notion that the neural encoding of reaching and grasping relies on multiple neurons to reduce the inherent noisiness of individual neurons. It also suggests that neural dimensions with task-related information are not systematically less noisy than other neural dimensions.

### Initial Partitioning versus Demixing: Implications for Overlap of Subspaces

In contrast to the recently developed approach of demixed PCA (dPCA), which identifies PCs that best transform the original data to reconstruct different partitions of the neural activity (Brendel et al., 2011; Kobak et al., 2016), we chose to partition the data first and then perform standard PCA on each partition separately. Because any dimension of the neural space may have variance in more than one partition, dPCA may not always identify the highest variance PCs in a given partition if those PCs also include variance from other partitions. While dPCA may improve visualization, our present approach was designed to quantify the magnitude and dimensionality of neural activity in each partition separately, as well as the overlap between partitions. We also performed dPCA on our dataset with qualitatively similar results (not illustrated). The demixed PCs were more distinct from those of other partitions, but a larger number of demixed PCs were required to account for the same amount of variance within a partition. These differences were to be expected because our separate PCA of each partition identified the optimal orthogonal transform to account for the most variance in the fewest dimensions of a given partition without regard for other partitions.

Partitioning first, we found a moderate amount of overlap be-tween the GT and LO dimensions, more than would be expected by chance alone. In contrast, a recent study of reaching movements found that the condition-invariant and condition-dependent partitions were almost completely orthogonal (Kaufman et al., 2016). This study, however, used a form of dPCA that con-strained the identified dimensions to be orthogonal (Brendel et al., 2011), which could have minimized any overlap. Moreover, the grasps and manipulations included in our RGM task may have required activity in larger numbers of neural dimensions, providing more opportunity for overlap of the present GT (condition-invariant) and LO (condition-dependent) partitions.

### Population Dynamics in the GT and LO Partitions

The neural trajectory in the GT partition was more than a simple rise and fall along a single dimension in the neural state space.The GT neural trajectory rotated through approximately four dimensions over the time course of an RGM trial. Such rotation of neural trajectories has been described previously in cortical neuron populations under a variety of circumstances (Churchland et al., 2012; Hall et al., 2014; Kaufman et al., 2014; Michaels et al., 2016). Our results show that a single major rotation in a low-dimensional manifold occurs not only with reaching movements but also when the movement includes grasping and manipulation.

The neural dimensions related to the LO features of the task shifted progressively over the time course of RGM trials. While previous studies have shown that much of such condition-dependent activity during reaching tasks can be captured by a single oscillation within a neural plane identified with jPCA (Churchland et al., 2012), we observed more complex trajectories applying jPCA to the LO partition in the present RGM task (Figure S7). For an ideal cyclic trajectory to occur in a single plane, the neural activity should lie in a given neural dimension, move to an orthogonal neural dimension, shift back to the (negative) original dimension, and finally return to the (negative) second dimension before completing the cycle. Our results did not meet this pattern in two ways (Figure 6). First, the neural dimensions occupied early during RGM movements were not substantially reoccupied later following a period of orthogonal activity (overlap = 0). Instead, the neural dimensions of LO activity early versus later became progressively more distinct, approaching a random orientation with respect to one another. Second, the variance in the LO dimensions occupied substantially more of the neural space than a single, dominant plane. As the complexity of various movements increases—such as adding grasping and manipulation to reaching—our findings suggest that the percentage of condition-dependent variance accounted for in a single 2D neural plane may diminish, possibly becoming even less than in the present study as the set of movements examined become more varied and naturalistic.

### Shifting Task Encoding with Time

Several previous studies have highlighted the evolution of individual neuron tuning as a movement evolves in time, showing shifts between encoding of movement direction, distance, and target (Fu et al., 1995); tuning to particular kinematic trajectory fragments (Hatsopoulos et al., 2007); or changes in directional tuning (Churchland and Shenoy, 2007; Suway et al., 2018); or limb biomechanics (Suminski et al., 2015). Rather than encoding within a set of neural dimensions fixed across time (as would be expected if each neural dimension represented a weighted, linear representation of a given movement parameter), the pre-sent time-resolved PCA showed that the neural dimensions with the most LO activity shifted progressively over the time course of RGM trials. A large majority of the present task-related units showed time-varying tuning to both location and object (Rouse and Schieber, 2016a). Hence it was the combination of M1 neurons carrying LO information—not different sets of neurons encoding either location or object—that shifted gradually over the movement time course. A similar progressive shift has been described in the sub-population of parietal area 7a neurons carrying task-critical spatial information (Crowe et al., 2010).

Although reaching and grasping traditionally have been considered to be mediated through independent channels (Jeannerod, 1984), considerable evidence suggests that under appropriate conditions, variation in reaching affects grasping and vice versa (Wallace and Weeks, 1988; Paulignan et al., 1991; Hoff and Arbib, 1993; Haggard and Wing, 1998; Connolly and Goodale, 1999). Our previous studies of the present RGM movements have shown that location-related variance predominates early and object-related variance later, not only in the activity of individual M1 neurons (Rouse and Schieber, 2016a) but also in the EMG activity of individual muscles (Rouse and Schieber, 2016b) and in the kinematics of individual joints (Rouse and Schieber, 2015). Our findings here that a similar population of neurons in the motor cortex is related both to reach location and to the object grasped and manipulated—but with progressively shifting neural dimen-sions of activity—may provide a framework to better understand these observations. We note, however, that our neural recordings sampled the upper extremity representation in new M1 (Rathelot and Strick, 2009) in the anterior bank and lip of the central sulcus, and our findings therefore may not necessarily apply to “old” M1 on the crown of the precentral gyrus.

### Limitations in Understanding Natural Motor Behavior

The concepts of GT, condition-dependent, and noise variance are defined within the framework of repeated trials between which experimental conditions are varied. Both the reaching movements studied by others and the RGM movements studied here involved a clear beginning and end. In part, the GT activity may reflect this trial structure. Whether GT (condition-independent) and LO (condition-dependent) activity are so distinct in behaviors without well-defined movement onsets and offsets remains un-clear. More naturalistic forelimb movements will need to be studied to assess the extent to which neural trajectories in the GT partition maintain their relatively simple rotational dynamics or become more complex (Hall et al., 2014). Likewise, more diverse movement conditions will be needed to assess how many different dimensions of the neural space are occupied in making the diverse arm and hand movements possible in primates.

### Implications for Neural Control of Movement and Decoding

While encoding task-specific information with activity in gradually shifting neural dimensions may entail more computational complexity than time-invariant encoding, we speculate that the progressive shifting of the active dimensions in the LO partition together with the rotational dynamics in the GT partition could better encode the temporally varying patterns of muscle activity and kinematics needed across the time course of RGM movements. Shifting neural subspaces could provide selective encoding of specific kinematic, dynamic, or muscular parameters most critical at specific times, leading to more precise control of movements on different scales, including both the quick reach to a target and the fine manipulation of the object in our RGM task. These considerations may be relevant to the design of brain-computer interfaces, which are limited to recording from a small fraction of all neurons, each individual neuron having a limited dynamic range. Indeed, recent work has suggested that shifts in neurons’ dynamic ranges already occur in brain-computer interface movements when switching between a simple 2D and 3D cursor task (Rasmussen et al., 2017). Our findings further suggest that control of upper extremity brain-computer interfaces might be improved by decoding strategies that make use of shifting active neural dimensions.

## STAR*METHODS

### CONTACT FOR REAGENT AND RESOURCE SHARING

Further information and requests for resources and reagents should be directed to and will be fulfilled by the Lead Contact, Marc H. Schieber (mschiebe@ur.rochester.edu).

### EXPERIMENTAL MODEL AND SUBJECT DETAILS

#### Non-human primates

Two male rhesus monkeys, L and X (weight 9 and 12 kg, age 7 and 9 years old, respectively), were subjects in the present study. All procedures for the care and use of these nonhuman primates followed the Guide for the Care and Use of Laboratory Animals and were approved by the University Committee on Animal Resources at the University of Rochester, Rochester, New York. The neuronal recordings analyzed here were previously published in Rouse and Schieber (2016a).

### METHOD DETAILS

#### Behavioral task

Each monkey was trained to perform a RGM task as described in detail previously (Rouse and Schieber, 2015). Briefly, four objects were placed in the coronal plane in front of the subject: a perpendicular cylinder, a coaxial cylinder, a push button, and a sphere. The four objects were located at 45° intervals on a semicircle centered on a second coaxial cylinder that served as the home object from which all trials were initiated. The array of peripheral objects could be rotated about the home object to vary the combination of reach location and object type. Objects thereby were positioned in 1 of 8 different locations ranging from 0° (to the monkey’s right on the horizontal meridian) to 157.5° (to the left, 22.5° above the horizontal meridian) in steps of 22.5°. Data was collected in blocks with the objects in a fixed location for approximately 10 successful trials involving each object. Then the peripheral object array was rotated to a different position. Due to physical constraints, only 24 of the 32 potential LO combinations were used. In each recording session, approximately 30 trials were completed to each of these 24 LO combinations.

#### Neural recordings

Six 16-channel floating microelectrode arrays (MicroProbes) were implanted in the anterior bank of the central sulcus to record from M1 in each monkey, using methods described in detail previously (Mollazadeh et al., 2011; Rouse and Schieber, 2016a). In both animals, intracortical microstimulation (ICMS) of some electrodes on the most lateral array generated twitches in facial musculature, whereas ICMS of some electrodes on the most medial array generated twitches of trunk musculature, with many sites in between generating twitches of shoulder, elbow, wrist, or digit musculature (Figure 1 in Rouse and Schieber, 2016a), altogether indicating that the present recordings spanned the entire upper limb representation of M1 in a similar way for both monkeys. Channels with spiking activity were thresholded manually on-line. The spike snippets were sorted offline with a custom, semi-automated algorithm (Rouse and Schieber, 2016a). Both single- and multi-unit recordings were used in the present analysis. The current datasets consist of approximately 1/3 definite single units, 1/3 probable single units with minor contamination, and 1/3 multi-units (Rouse and Schieber, 2016a).

### QUANTIFICATION AND STATISTICAL ANALYSIS

#### Data analysis

The firing rate of each unit was estimated by convolving spike times binned at 1 ms resolution with a Gaussian (s = 50 ms) smoothing window. All data was time-aligned separately on four behavioral events present in each successfully performed trial: instruction onset (I), movement onset (M), contact with the peripheral object (C), and beginning of the final hold (H). (Movement onset was defined based on the sudden increase in the average speed of 36 markers positioned on the upper extremity tracked at 200 Hz by a Vicon motion capture system [Rouse and Schieber, 2015].) All firing rates were square-root transformed. As firing rates tend to be distributed closer to what would be expected for a Poisson process than a normal distribution, the square-root transformation renders variance more similar from low to high firing rates, making the comparison of different rates more reliable (Kihlberg et al., 1972; Ashe and Georgopoulos, 1994). The mean square-root transformed firing rate across all trials at the time of instruction onset (I) was considered to be the baseline firing rate for each unit. This baseline firing rate was subtracted from all firing rates so each unit’s firing rate started with zero mean at time point I. These smoothed, square-root transformed, baseline-subtracted firing rates, *r*, were used for all analyses. Notably, as shown in the Results, after square-root transformation and baseline subtraction, noise was uniform in magnitude throughout individual trials (see Figure 1) and was distributed evenly across units (see Figure 2), consistent with the theoretical assumptions of normality and uniform scaling of variables required for principal component analysis.

The complete dataset consisted of the estimated firing rate (*r*) in a three-dimensional array composed of: *N* spiking units x *I* trials x *T* time points. There were *N* = 346 and 466 sorted spiking units for monkeys L and X, respectively. Each session consisted of approximately 700 successfully performed RGM trials. For each monkey, there were *K* = 24 different trial types, i.e., LO combinations. Aligning separately on the four behavioral events—I, movement onset, peripheral object contact, and H—and truncating the data at the midpoint time between events using the median event durations for each monkey, there were *T* = 658 time points for monkey L and *T* = 706 time points for monkey X corresponding to 658 and 706 ms of firing rate data per trial.

#### Partitioning of Neural Modulation

To examine the variation in the neural trajectories that depended on the 24 LO conditions, we partitioned the firing rate modulation of each unit into GT modulation, LO modulation, and noise. As illustrated for an example unit in Figure 7, the GT modulation that occurred during all RGM movements was calculated first by averaging at each time point across all trials, regardless of the particular LO condition. This GT modulation, which can be considered to be the activity associated with performing any RGM movement in general, then was subtracted from the firing rate at each time point of each trial. The remaining firing rate modulation then was aver-aged at each time point across all trials for each of the 24 LO conditions separately, providing the time course of the unit’s modulation that depended on the particular LO condition. Finally, the GT and the appropriate LO modulation both were subtracted from the unit’s original activity in each trial, leaving the portion of the firing rate that could be attributed neither to the GT nor to the LO modulation, which we therefore considered to be “noise.” All three partitions—GT, LO, and Noise—thus were calculated for each unit as a function of time in each trial. Note, however, that the GT modulation of a given unit was identical across all trials, and the LO modulation was identical across all trials of each LO condition.

The deviation of each unit’s firing rate from its baseline at each time point, *t*, then can be expressed as:

$$r\left(t\right)=r_{GT}\left(t\right)+r_{LO,k}\left(t\right)+r_{Noise,i}\left(t\right)$$

where *r*_*GT*_ (t) is the same for all trials regardless of location or object and varies only as a function of time; *r*_*LO*,*k*_ (*t*) is condition-dependent, differing for each of the *k* = 1…24 LO combinations; and *r*_*noise*,*i*_ (*t*) was calculated by subtracting both the GT modulation and the appropriate LO modulation from the original firing rate in each trial, *i.*

The sample variance was calculated for each unit across all trials partitioned into GT, LO, and Noise. The total variance, calculated as the sum of the squares (*r*^*2*^), is equal to the sum of the partitioned sum of the squares for the three partitions (*r*_*GT*_, *r*_*LO*_, *r*_*Noise*_):

$$\sum_{n}^{N}\sum_{i}^{I}r{\left(t\right)}^{2}=\sum_{n}^{N}\sum_{i}^{I}r_{GT}{\left(t\right)}^{2}+\sum_{n}^{N}\sum_{i}^{I}r_{LO,k}{\left(t\right)}^{2}\sum_{n}^{N}\sum_{i}^{I}r_{noise,i}{\left(t\right)}^{2}$$

where *N* is the total number of units indexed by *n* and *I* is the total number of trials indexed by *i*. The sum for each trial, *i*, has a fixed value for the GT, a particular value for the location & object, *k*, and individual value for noise on trial *i*. The sum of squares across trials then can be combined across all units to yield the total sum of squares across all units as a function of time.

For some analyses, the LO partition was partitioned further to calculate the variance of the individual factors: location, object, and their interaction (location x object).

$$\sum_{n}^{N}\sum_{i}^{I}r_{LO,k}{\left(t\right)}^{2}=\sum_{n}^{N}\sum_{i}^{I}r_{Loc}{\left(t\right)}^{2}+\sum_{n}^{N}\sum_{i}^{I}r_{Obj}{\left(t\right)}^{2}+\sum_{n}^{N}\sum_{i}^{I}r_{Loc\times Obj}{\left(t\right)}^{2}$$

This partitioning of the location, object, and interaction was identical to the time-resolved analysis of variance used previously (Rouse and Schieber, 2016a). Compared to a full 3-way ANOVA of time, location, and object, certain factors have been combined. The i) time-independent location and time x location interaction term and ii) time-independent object and time x object interaction terms were combined into single ANOVA terms we denote simply as location and object, respectively, as all location and object effects are assumed to vary with time (see Kobak et al., 2016 for further details).

#### Bootstrapping to Estimate Confidence Intervals of Neural Variance

The 95% confidence interval for the variance of the recorded neural population (Figure 1) was estimated using bootstrapping. A new distribution of variances of all spiking units was created by resampling with replacement from the original population, repeated 1000 times. The range of mean variances was estimated by using the 2.5^th^ and 97.5^th^ percentiles of the mean variances of these randomly resampled distributions.

#### Principal Component Analysis of Neural Partitions

The neural space which contained modulation for each partition was examined using principal component analysis (PCA). PCA identifies and ranks orthogonal neural dimensions from largest to smallest variance in the *N*-dimensional neural state space. Separate PCAs were performed on each of the three partitions of firing rate modulation.

1. GT modulation - PCA was performed on r_GT_ which consisted of *T* firing rates (averaged across all *I* trials) in *N* neural dimensions.
2. Location & object modulation – PCA was performed on r_LO_ which consisted of *K* ∙ *T* firing rates across *N* neuron dimensions, respectively.
3. Noise modulation – PCA was performed on r_noise_(t) which consisted of *I*∙ *T* firing rates across *N* neural dimensions.

Each PCA generates an orthonormal transformation matrix, *W*, that transforms the input firing rates, *r*, into a rotated space.

#### Quantifying subspace overlap

The overlap between two data partitions or the LO data at two time points was estimated as the similarity of the two datasets’ covariance matrices:

$$Overlap=\frac{tr\left(\Sigma_{1}\Sigma_{2}\right)}{{\left\|\Sigma_{1}\right\|}_{F}\cdot {\left\|\Sigma_{2}\right\|}_{F}}$$

The trace of the product of the two covariance matrices, *tr*(Σ_1_Σ_2_), is the sum of all dot products of all dimensions scaled by their variance. The result is then normalized by the total variance using the Frobenius norm (∥…*F*∥) of the covariance matrix to obtain a metric between 0 and 1.

The expected amount of overlap was simulated for two conditions: i) two subspaces sampled from a fixed, common space that does not change with time; and ii) two subspaces sampled at random with no relationship. To estimate how much overlap would be expected if a common space with a constant LO data distribution occurred across all time-points, 24 data points to simulate the 24 LO movements were drawn from a multivariate, normal distribution with the covariance of the LO data observed across all times. The overlap was then calculated from pairs of 100 randomly generated data points, (100×99) ÷ 2 = 4950 independent pairs, to estimate the 5th percentile, generating a lower bounds of the 95% confidence interval. To estimate how much overlap would be expected at random, 24 data points to simulate the 24 LO movements were drawn from a multivariate, normal distribution with the covariance of the LO data observed across all times, but the dimensions were randomly permuted so the pair of subspaces had no relationship. The overlap was then calculated from 4950 independent pairs to estimate the 2.5^th^ and 97.5^th^ percentile to generate a 95% confidence interval.

## Supplementary Material

1

2

## Figures and Tables

**Table:** KEY RESOURCES TABLE

REAGENT or RESOURCE	SOURCE	IDENTIFIER
Deposited Data		

Neuronal Recordings	Rouse and Schieber, 2016a	http://www.jneurosci.org/cgi/doi/10.1523/JNEUROSCI.1716-16.2016

Software and Algorithms		

MATLAB	Mathworks	https://www.mathworks.com/products/matlab.html
TEMPO Experimental Control System	Reflective Computing	http://reflectivecomputing.com/
Multichannel Acquisition Processor Software	Plexon Inc. Plexon	http://plexon.com/products/map-software
Offline Sorter	Plexon Inc. Plexon	https://plexon.com/products/offline-sorter

Other		

MAP Data Acquisition System Plexon	Plexon Inc. Plexon	https://plexon.com/products/map-data-acquisition-system-plexon/
Floating Microelectrode Arrays (FMAs)	Microprobes for Life Sciences	https://www.microprobes.com/products/multichannel-arrays/fma

## Highlights

- Neural modulation related to location and/or object is smaller than general task modulation
- Neural dimensions of location and/or object overlap with those of the general task
- Neural dimensions of location and/or object modulation shift as a trial progresses in time
- Noise is large but evenly distributed across neural dimensions and time
