# Supplementary material for: Condition-Dependent Neural Dimensions Progressively Shift during Reach to Grasp
Source: Cell Rep. Author manuscript; Available in PMC 2019 Feb 4. (PMC6361546; doi:10.1016/j.celrep.2018.11.057)
Supplement: 1 [file NIHMS1517195-supplement-1.pdf]

Cell Reports, Volume *25*

Supplemental Information

Condition-Dependent Neural Dimensions Progressively Shift during Reach to Grasp

Adam G. Rouse and Marc H. Schieber

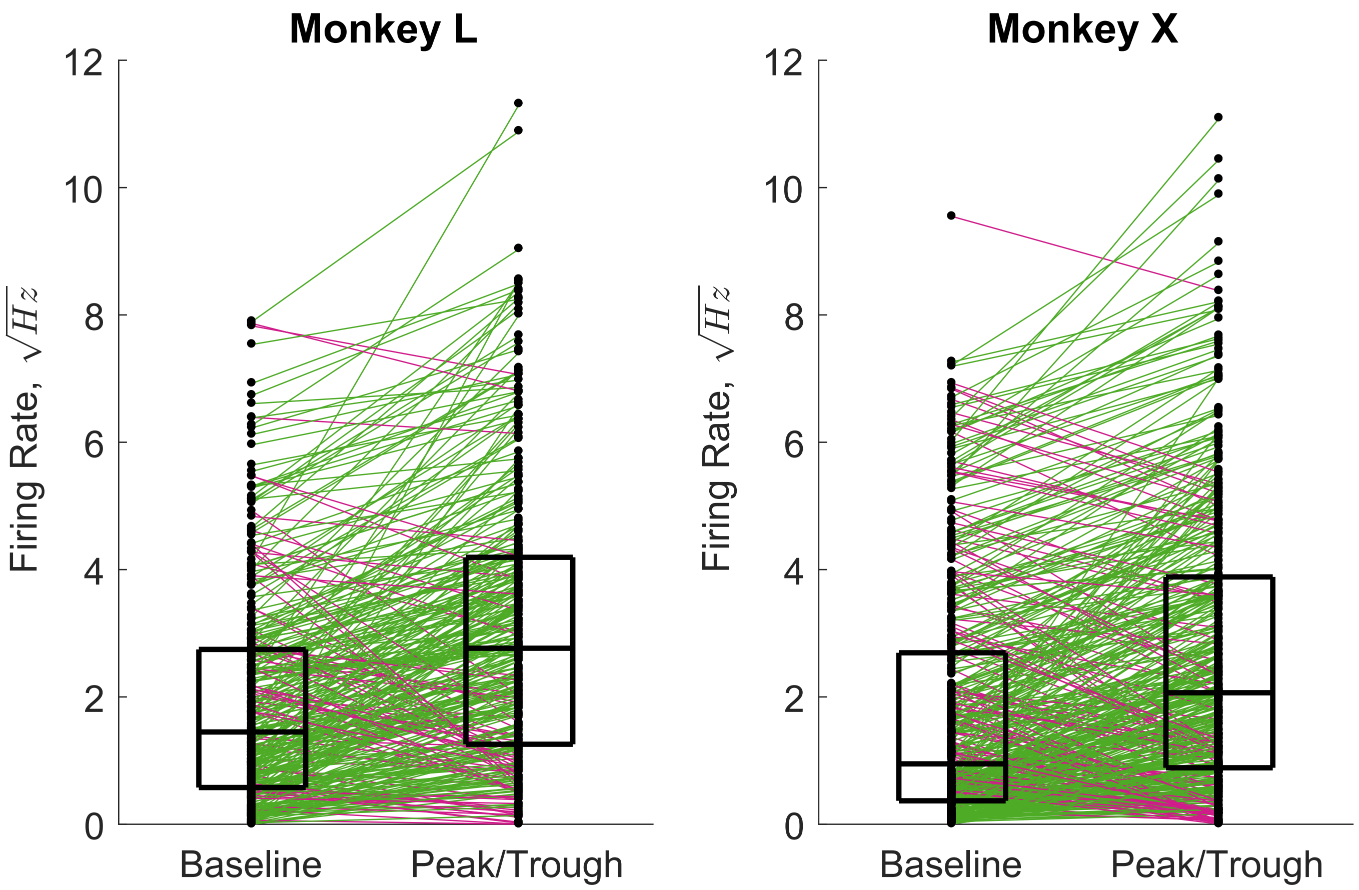

Figure S1. Changes in firing rate in the General Task Partition. Related to Figure 1. For each unit, the baseline firing rate of general task activity at the Instruction time point is compared to the peak or trough firing rate, i.e. the greatest deviation—increase or decrease—from baseline between the Instruction and the final Hold. Boxes indicate the 25th, 50th, and 75th percentiles. Firing rate increases are identified with green lines, decreases with magenta lines. For 282/346=82% (monkey L) and 364/466=78% (monkey X) of units, the largest deviation was greater than baseline (i.e. a peak) while the remaining deviations were less than baseline (i.e. a trough). For all subsequent analyses, the baseline firing rate was subtracted from all firing rates throughout the trial.

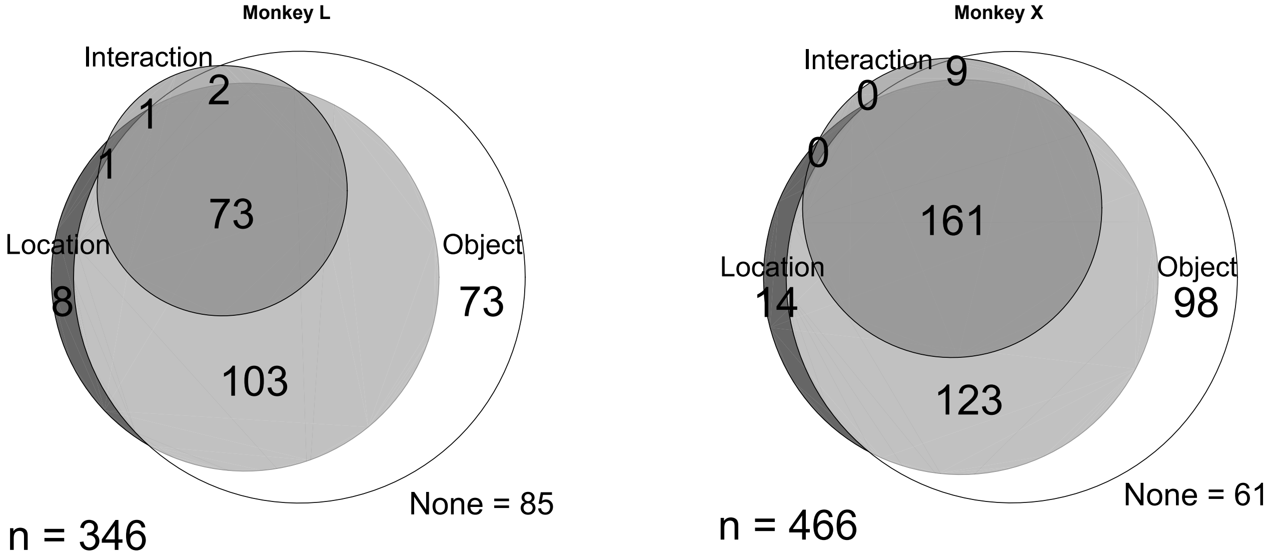

Figure S2. The number of units significantly tuned to Location, Object, and/or Interaction (Location x Object). Related to Figure 1. Significant effects were identified using two-way ANOVA with Location and Object as factors. ANOVA was performed on each time point individually from Instruction until Final Hold, and p-values were Bonferroni corrected for the number of time points x 3 tests (Location, Object, Interaction) using methods described in detail in Rouse & Schieber (2016a).

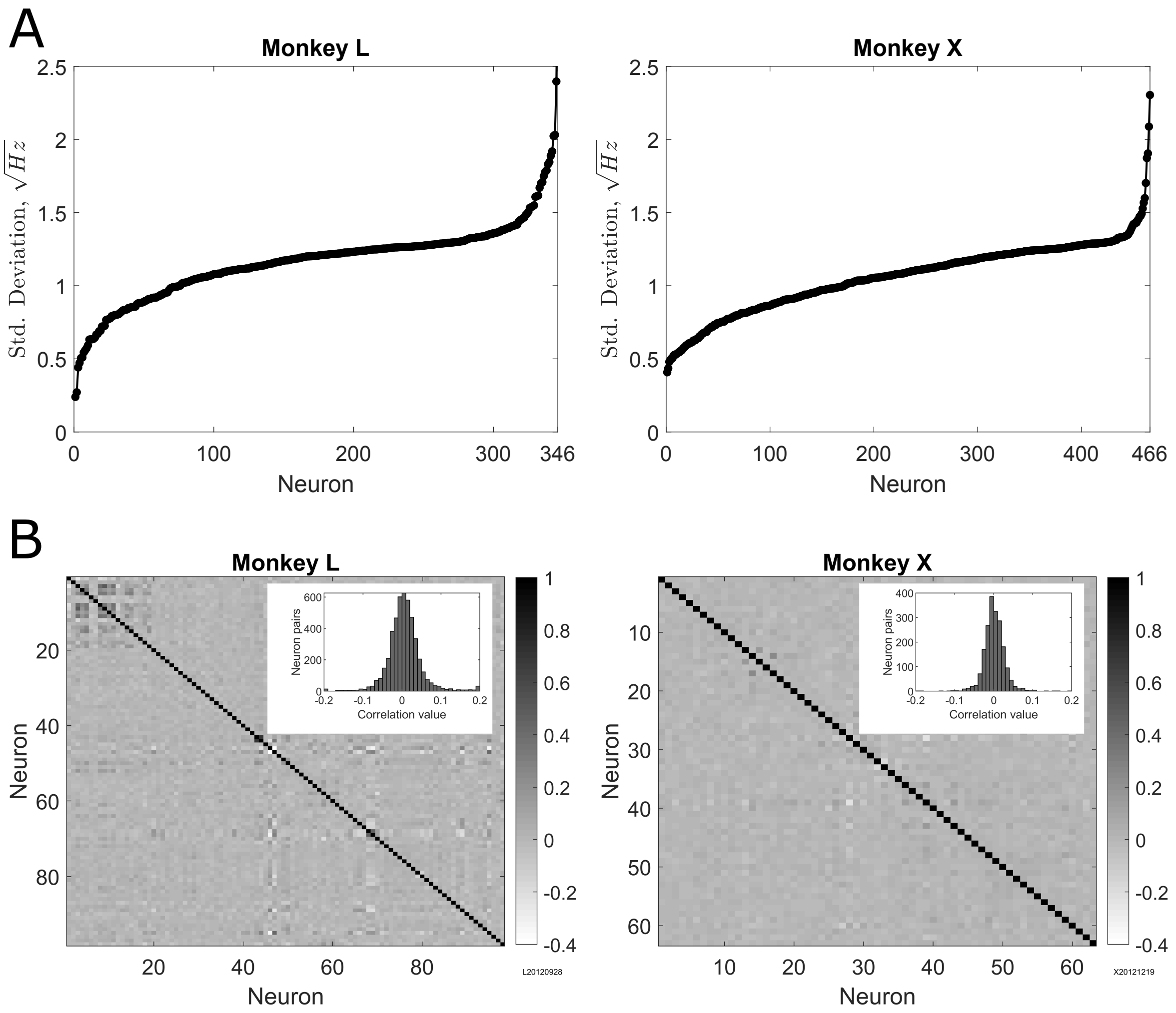

Figure S3. Noise Analysis. Related to Figure 2. Noise activity was examined using the residual firing rates obtained after subtracting GT and LO activity. A) The rank-ordered standard deviations of the residual square-root transformed firing rates for all units are shown for each monkey. These standard deviations ranged from 0.24-3.24 $\sqrt{Hz}$ with a majority, 82% (monkey L) and 81% (X), of units between 0.8-1.4 $\sqrt{Hz}$. B) Correlation coefficients between residual firing rates for all pairwise combinations of units in each monkey in a single recording session are shown as a grayscale matrix, with the inset showing the corresponding histogram of correlation coefficients for all neuron pairs. Noise correlations between units were small: Correlation coefficients had mean absolute values of 0.03 (L) and 0.02 (X), and 96% (L) and 99% (X) of neuron pairs had correlation coefficients between -0.1 and +0.1. These small noise correlations are consistent with previous pair-wise analyses that observed the mean noise correlation between neurons pair was slightly positive, ranging from 0.02-0.04 depending on time interval (Lee et al., 1998). Both the relatively uniform standard deviations across units and low magnitude pairwise correlations suggest the noise activity unexplained by the experimental variables can be reasonably well fit with a uniform, independent noise model.

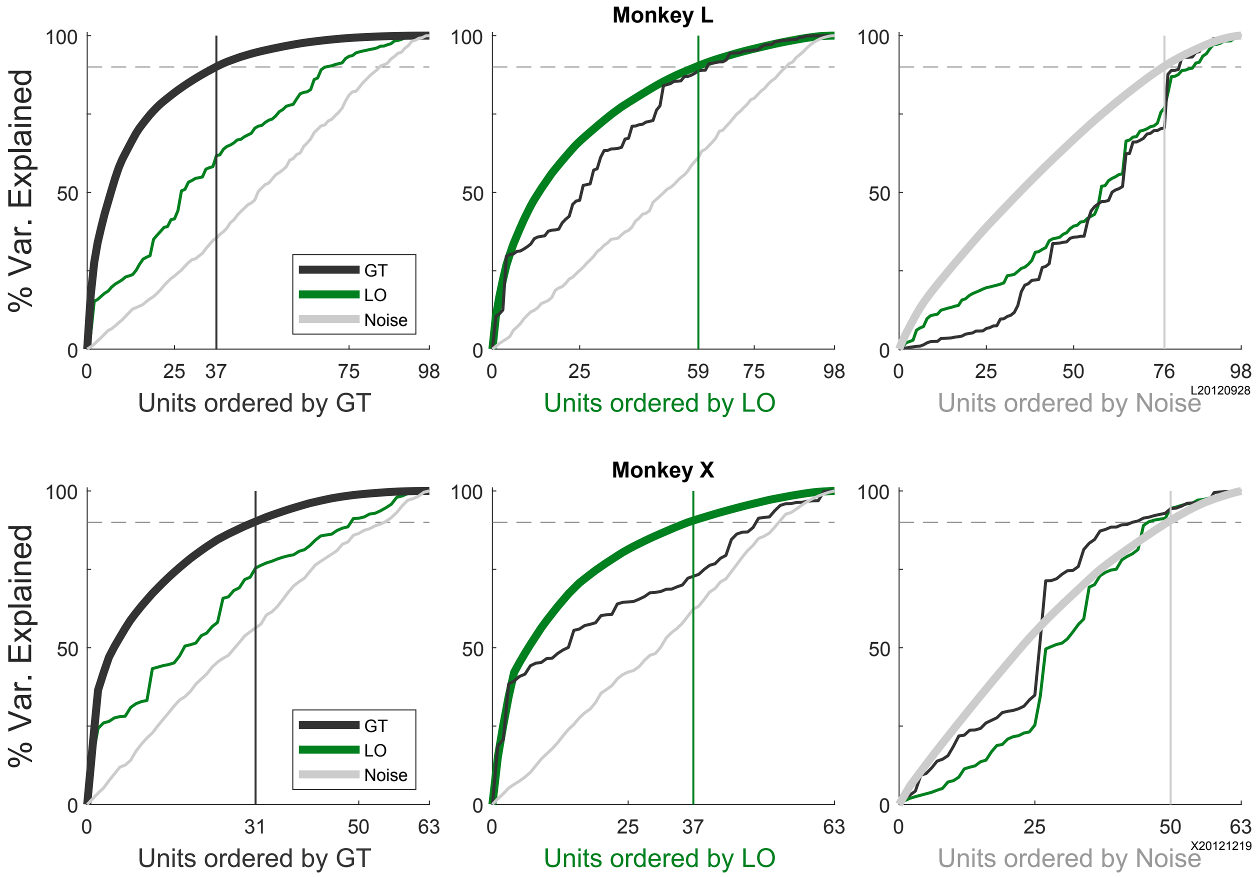

Figure S4. The percentage of variance explained by individual spiking units. Related to Figure 2. Without any dimensionality reduction, the individual units were ordered according to the magnitude of their individual firing rate variance in a given partition. In each panel, the thick line shows the cumulative variance explained by individual units ordered by their individual variance in that partition: GT (left), LO (center), and Noise (right) partitions. The cumulative variance explained by those ordered units in each of the other two partitions has been plotted as thin lines. To account for ≥90% of the GT variance required 37 units in monkey L and 31 units in monkey X. To account for ≥90% of the LO variance required 59 units in L and 37 units in X. To account for ≥90% of the Noise variance required 76 units in L and 50 units in X. The neural encoding of GT and LO thus was distributed across a large fraction of the M1 population in each monkey. Note, only simultaneously recorded data from a single recording session is used since this analysis relies on noise variability of individual trials.

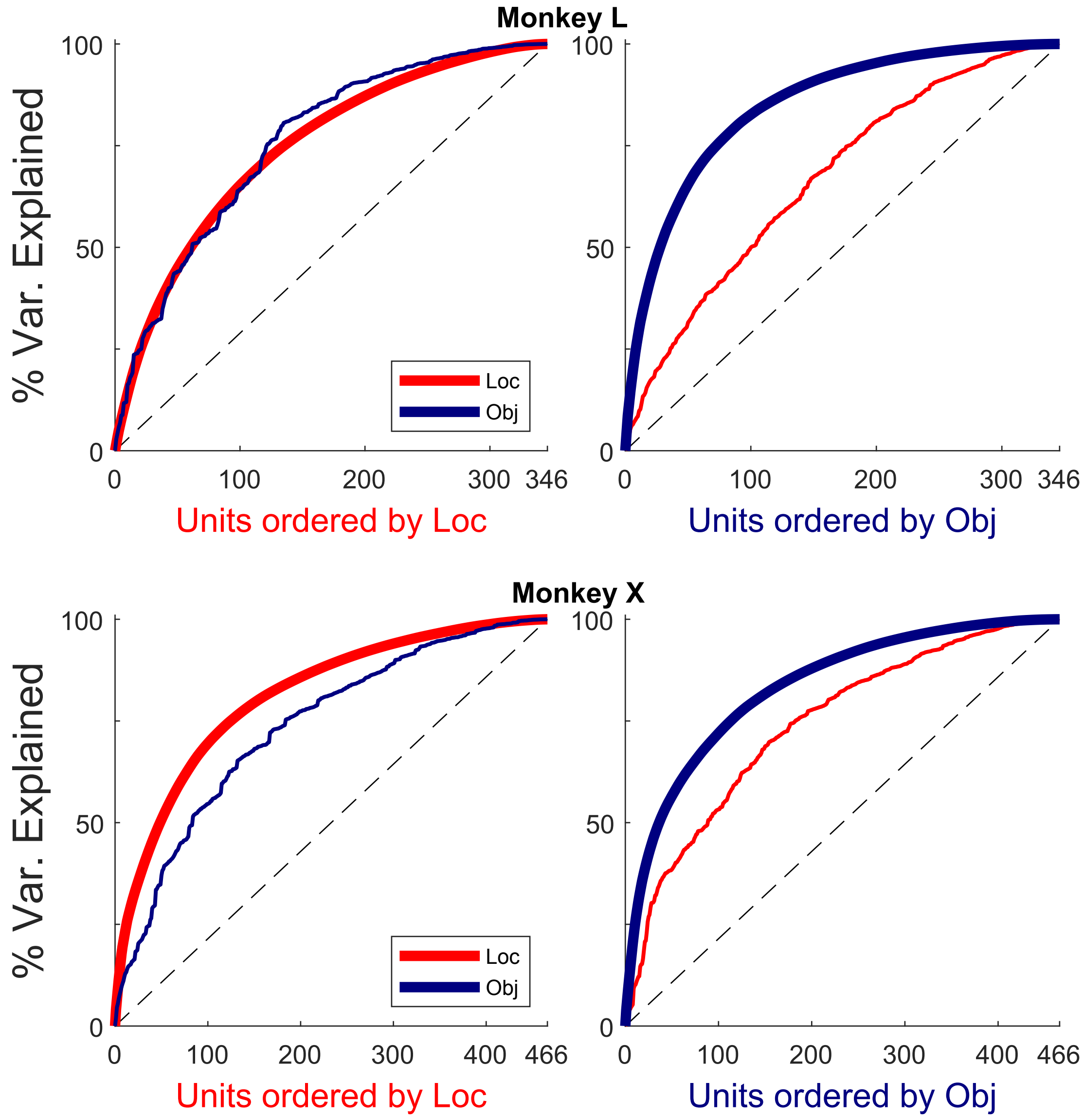

Figure S5. The location and object variance explained by individual spiking units. Related to Figure 2. The units have been ordered according to their individual variance explained by location (left panel) or by object (right panel), with the thick line showing the cumulative variance. The thin line shows the variance explained by those ordered units for the other factor: object (left panel) or location (right panel). For both animals, units with larger location variance were more likely to have larger object variance and vice versa, as evidenced by the thin colored lines lying entirely above the black dashed line.

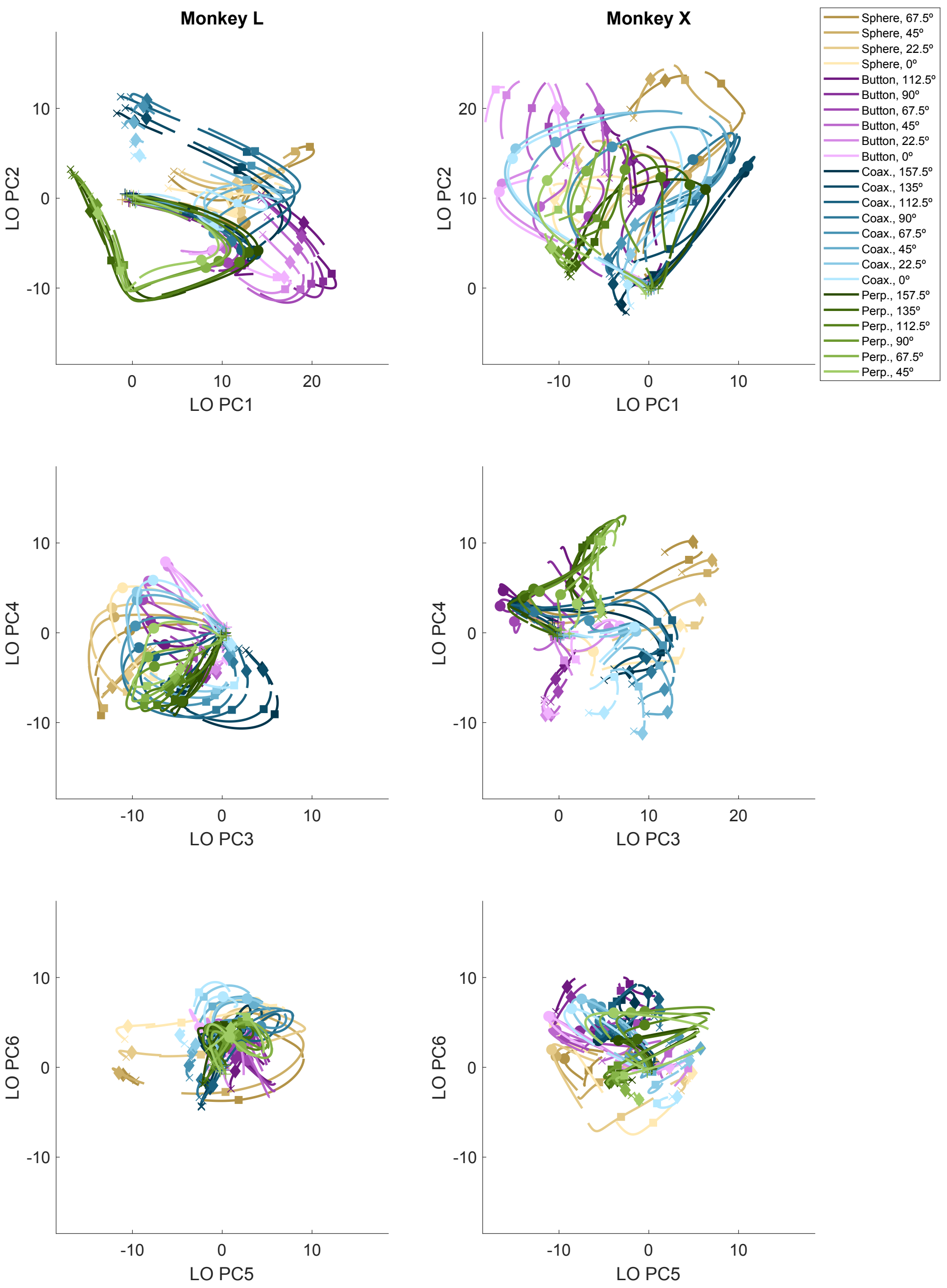

Figure S6. PCA of LO partition. Related to Figure 4. PCA was performed on the LO partition across all times. The average firing rate for each of the 24 LO conditions has been plotted in the first six PCs for each animal. No simple axis of separation for location and/or object was observed across all times. In addition, the maximum variance occurred at different time points, suggesting the neural dimensions with the most LO variance shifted with time. These first six PCs accounted individually for 26% to 4% (from PC1 to PC6 in monkey L) and from 23% to 5% (X), and cumulatively for 63% (Monkey L) and 68% (Monkey X), of the total LO variance. Data have been aligned separately at the times of Instruction onset (+), Movement onset (circle), Contact (square), and Hold (diamond).

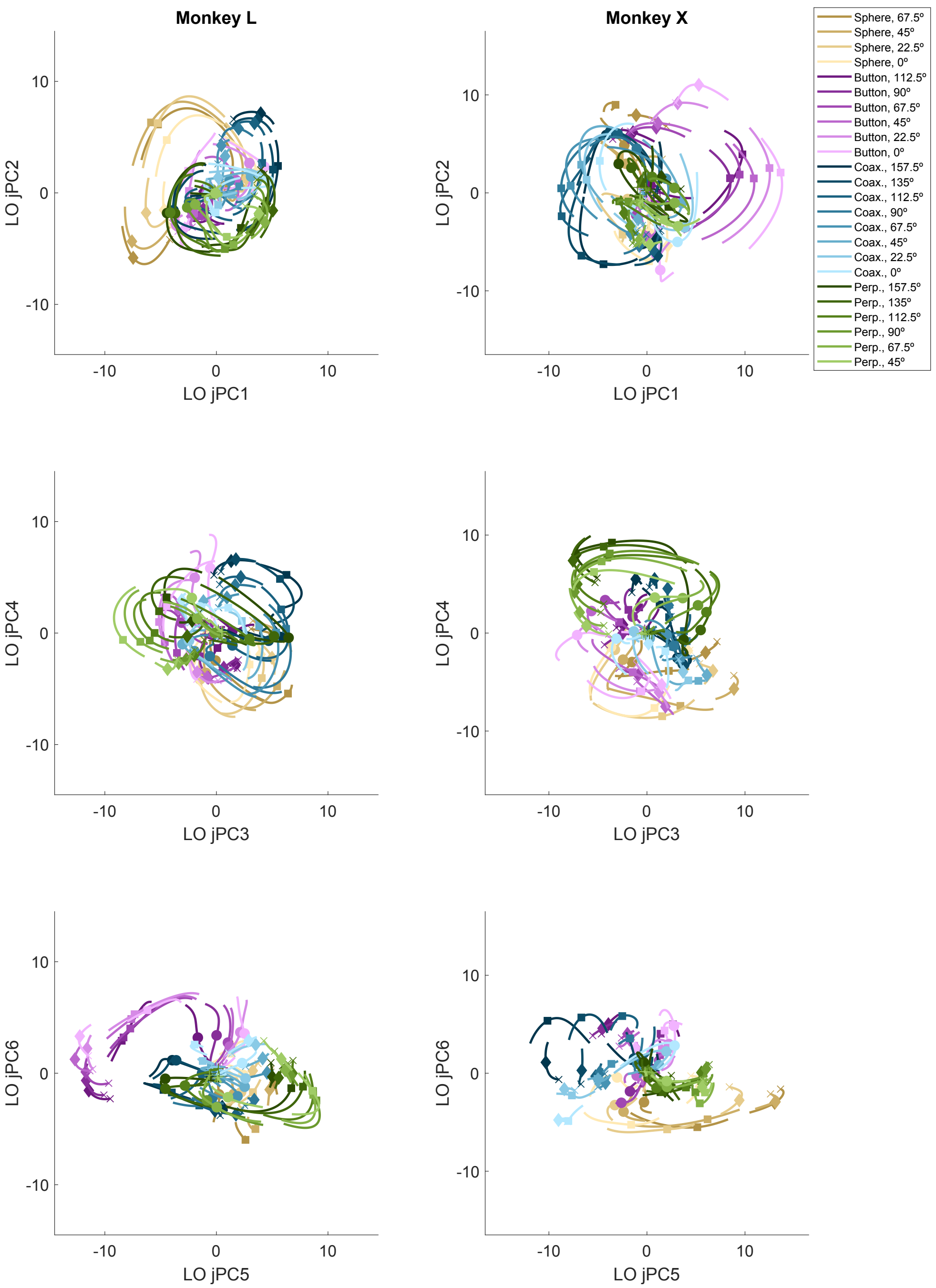

Figure S7. jPCA of LO partition. Related to Figure 4. jPCA was performed on the LO partition following the methods of Churchland et al. (2012). The neural trajectories for the 24 LO conditions have been plotted in the first 3 jPC planes, which have the most rotational activity. In some planes rotational trajectories are apparent for most movements, while in others substantial rotational activity appears for only a subset of the 24 LO conditions. The magnitude of LO variance was similar in all three planes: 20%, 14%, and 11% in Monkey L; 16%, 15%, and 13% in Monkey X for jPC planes 1 vs 2, 3 vs 4, and 5 vs 6, respectively. This observation suggests that a single dominant, rotational plane failed to account for most of the LO activity. Data have been aligned separately at the times of Instruction onset (+), Movement onset (circle), Contact (square), and Hold (diamond). Note that the same square-root transform of firing rates (used in all other analyses in the present paper) was applied prior to jPCA and not the normalization procedures used by Churchland et al.
